# Evaluation of Depression in Adults With and Without Type 2 Diabetes Mellitus: A Comparative Study

**DOI:** 10.7759/cureus.113389

**Published:** 2026-07-26

**Authors:** Muhammad Hamza, Saifullah Chan, Muhammad Usman Sharif, Naveed Ahmed, Ayesha Sharif, Syed Asadullah Agha, Zaid Khan

**Affiliations:** 1 Internal Medicine, Ayub Teaching Hospital, Abbottabad, PAK; 2 Pulmonology, Ayub Teaching Hospital, Abbottabad, PAK; 3 Internal Medicine, Pakistan Institute of Medical Sciences, Islamabad, PAK; 4 Internal Medicine, Rawalpindi Medical University, Rawalpindi, PAK; 5 Medicine and Surgery, Lady Reading Hospital, Peshawar, PAK

**Keywords:** cross-sectional study, depression, mental health, pakistan, phq-9, type 2 diabetes mellitus

## Abstract

Background: Depression is a common psychiatric disorder frequently associated with type 2 diabetes mellitus (T2DM) and negatively impacts quality of life and disease outcomes.

Objective: The objective of this study is to compare the frequency and severity of Patient Health Questionnaire-9 (PHQ-9)-screened clinically significant depressive symptoms among adults with T2DM and non-diabetic adults.

Methodology: This cross-sectional comparative study was conducted from August 2025 to April 2026. A total of 114 participants were enrolled using non-probability consecutive sampling and divided equally into T2DM (n = 57) and control (n = 57) groups. Depression was assessed using the PHQ-9 questionnaire, with a score ≥10 indicating clinically significant depression. IBM SPSS Statistics for Windows, Version 25 (Released 2017; IBM Corp., Armonk, New York, United States) was used to analyze the data, and the chi-square test was used for categorical variables.

Results: Depression was significantly higher in the T2DM group compared to controls. In diabetic patients, 61.40% (n = 35) were depressed, while 38.60% (n = 22) were not depressed. In the control group, 33.33% (n = 19) were depressed, and 66.67% (n = 38) were not depressed (p = 0.003). Depression was more frequent in participants aged 46-50 years and showed significant associations with gender and duration of diabetes. However, no significant difference was observed in depression severity distribution between the two groups.

Conclusion: Depression is significantly more prevalent among patients with T2DM compared to non-diabetic individuals.

## Introduction

Depression is a prevalent and debilitating mental disorder with symptoms that include low mood, anhedonia, and cognitive and somatic symptoms that affect functioning significantly over a prolonged period [1,2]. It is known to be a significant burden of disease worldwide and to be linked to reduced productivity, poorer quality of life, and increased healthcare utilization in all age groups and socio-economic groups [3,4]. Depression often occurs alongside chronic medical conditions, and it can have a negative impact on the outcome of these conditions, treatment compliance, and prognosis [5].

Type 2 diabetes mellitus (T2DM) is one of the most common chronic metabolic disorders in the world, which is defined by insulin resistance and a gradual loss of β-cell function [6]. Diabetes is a chronic condition, and the lifestyle changes that are required, the burden of medications, and the fear of complications create a significant psychological burden for those with diabetes [7,8].

There is a large volume of evidence that suggests there is a very strong bi-directional relationship between depression and T2DM [9]. Diabetes-related lifestyle modification, glycemic monitoring burden, and complications increase the risk of developing depressive symptoms [10,11]. Depression, on the other hand, can negatively impact glycemic control by decreasing self-care behaviors, physical activity, unhealthy eating habits, and neuroendocrine dysfunction, leading to worse metabolic outcomes [12].

Although much research has been conducted around the world, the prevalence and severity of depression in the diabetic population vary from one setting to another. Lack of standardized screening data and psychological morbidity under-recognition in the diabetic care settings are important challenges in low- and middle-income countries, including Pakistan. In addition, there are few studies that directly compare the severity of depression in diabetic and non-diabetic adults in the same clinical setting, using validated psychometric tools. This leaves a gap in the literature about the actual, as opposed to the general population, burden of depression among patients with T2DM. Thus, it is critical to assess depression in both diabetic and non-diabetic populations with standardized assessment instruments to identify differences in prevalence and severity patterns. This study was conducted to compare the prevalence of depression between adults with T2DM and non-diabetic adults and to compare depression severity between the two groups.

## Materials and methods

Study settings

The study was conducted at the Outpatient Department of Ayub Teaching Hospital, Abbottabad, Pakistan, a tertiary care teaching hospital that serves a large and diverse patient population from Abbottabad and the surrounding districts. As a hospital-based study, participants were recruited exclusively from the outpatient department; therefore, the study population represents adults attending this tertiary care facility rather than the general community population.

Study design and duration

The research was a comparative cross-sectional study carried out from August 2025 to April 2026.

Sample size and sampling technique

The required sample size was calculated using the OpenEpi sample size calculator for comparing two independent proportions. The calculation assumed a 95% confidence level (CI), 80% study power, a 1:1 ratio of participants between the T2DM and control groups, and an expected prevalence of depression of 60% among adults with T2DM and 30% among non-diabetic controls based on previous literature, resulting in a minimum required sample size of 57 participants per group (114 participants in total). Participants were subsequently recruited using a non-probability consecutive sampling approach from the outpatient department of Ayub Teaching Hospital, Abbottabad. Consecutive eligible participants were enrolled until the required sample size was achieved, comprising 57 patients with T2DM (Group A) and 57 non-diabetic controls (Group B).

Inclusion and exclusion criteria

Adults aged 30-50 years, irrespective of sex, with a confirmed diagnosis of T2DM for at least six months, verified through medical records and current treatment history, were eligible for inclusion in the T2DM group. The control group comprised age- and sex-comparable adults attending the same outpatient department who had no previous physician diagnosis of diabetes mellitus, no documented history of glucose-lowering medication use, and no evidence of diabetes recorded in their medical records. All participants were required to provide written informed consent and be able to complete the study questionnaire independently.

Individuals with a previously diagnosed psychiatric disorder or receiving psychiatric treatment were excluded to minimize potential bias in depression assessment. Participants with major comorbid medical conditions, including hypertension, chronic kidney disease, liver cirrhosis, stroke, or other endocrine disorders (except T2DM), as well as those with a history of substance or alcohol abuse, were also excluded to reduce potential confounding. Clinical variables such as HbA1c, fasting blood glucose, diabetes treatment modality (including insulin use), diabetes-related complications (e.g., neuropathy, retinopathy, and nephropathy), hypoglycemic episodes, and diabetes-related distress were not collected and were therefore not used as eligibility criteria or included in the analyses. Furthermore, biochemical screening (e.g., fasting plasma glucose or HbA1c) was not performed to confirm the absence of diabetes among control participants; diabetes status in the control group was determined based on documented medical history and medication records.

Data collection procedure

A structured questionnaire (see the Appendices) was developed by the research team and reviewed by departmental experts to ensure its relevance, clarity, and alignment with the study objectives. Written informed consent was obtained from all participants before data collection. Demographic and clinical information, including age, sex, place of residence, educational level, socioeconomic status, occupation, and body mass index (BMI), was recorded using the structured questionnaire. The diagnosis of T2DM was confirmed through participants' medical records and current medication history. Clinical variables such as HbA1c, fasting blood glucose, diabetes treatment modality, insulin use, and diabetes-related complications were not collected as part of the present study. Depressive symptoms were screened using the validated Patient Health Questionnaire-9 (PHQ-9) [13,14], which has also been used previously in Pakistani patients with T2DM to assess the frequency and severity of depression [15]. A PHQ-9 score of ≥10 was considered indicative of clinically significant depressive symptoms requiring further clinical assessment, consistent with established recommendations. Participants with PHQ-9 scores ≥10 were categorized according to symptom severity as moderate (10-14), moderately severe (15-19), or severe (20-27). No clinical psychiatric interview was performed to establish a definitive diagnosis of depressive disorder. Medical records and current medication history were reviewed to confirm the diagnosis of T2DM in the diabetic group. For the control group, the absence of diabetes was established by reviewing medical records to confirm no previous physician diagnosis of diabetes mellitus and no history of glucose-lowering medication use. Biochemical screening using fasting plasma glucose or HbA1c was not performed for the control group.

Data analysis

Data were analyzed using IBM SPSS Statistics for Windows, Version 25 (Released 2017; IBM Corp., Armonk, New York, United States). Continuous variables were assessed for normality and are presented as mean ± standard deviation (SD), while categorical variables are presented as frequencies and percentages. Baseline characteristics between the T2DM and control groups were compared using the independent-samples t-test for continuous variables and the chi-square (χ²) test for categorical variables.

To evaluate the independent association between T2DM and clinically significant depressive symptoms (PHQ-9 score ≥10), multivariable binary logistic regression analysis was performed. Potential confounding variables, including age, sex, BMI, place of residence, educational level, socioeconomic status, employment status, and duration of diabetes, were entered into the model and controlled for during the analysis. The results are presented as adjusted odds ratios (aORs) with 95% confidence intervals (95% CIs). A two-sided p-value <0.05 was considered statistically significant.

Ethical considerations

The study was carried out after receiving ethical permission from Ayub Medical College's Institutional Ethical Review Committee. Prior to data collection, all participants provided written informed permission, and the study maintained patient data confidentiality and anonymity.

## Results

A total of 114 participants were included, with equal distribution in the T2DM (n = 57) and control (n = 57) groups (Table 1). Male participants comprised 31 (54.39%) in the T2DM group and 29 (50.88%) in the control group, while female participants accounted for 26 (45.61%) and 28 (49.12%), respectively. Most participants resided in urban areas, and the distributions of education, socioeconomic status, and employment status were similar between the two groups. Age, gender, residence, education, socioeconomic status, and employment status did not differ significantly between groups (all p > 0.05). However, participants with T2DM had a significantly higher mean BMI than controls (27.84 ± 4.12 vs. 25.91 ± 3.78 kg/m², p = 0.018). Because BMI differed significantly between groups and may act as a potential confounder, it was included as a covariate in the multivariable logistic regression analysis.

**Table 1:** Study Participants' Clinical and Demographic Characteristics (n = 114) Values are presented as mean ± standard deviation or n (%). t: Independent-samples t-test; χ²: chi-square test; BMI: body mass index; SD: standard deviation.

Variable	Category	T2DM (n=57)	Controls (n=57)	Test value	p-value
Age (years)	Mean ± SD	42.36 ± 6.21	41.28 ± 6.05	t = 0.96	0.342
BMI (kg/m²)	Mean ± SD	27.84 ± 4.12	25.91 ± 3.78	t = 2.42	0.018
Gender	Male	31 (54.39)	29 (50.88)	χ² = 0.14	0.710
	Female	26 (45.61)	28 (49.12)		
Residence	Urban	34 (59.65)	32 (56.14)	χ² = 0.14	0.708
	Rural	23 (40.35)	25 (43.86)		
Education	Primary	18 (31.58)	16 (28.07)	χ² = 0.89	0.642
	Secondary	20 (35.09)	22 (38.60)		
	Higher secondary	19 (33.33)	19 (33.33)		
Socioeconomic status	Low	22 (38.60)	20 (35.09)	χ² = 0.62	0.731
	Middle	25 (43.86)	26 (45.61)		
	High	10 (17.54)	11 (19.30)		
Employment status	Employed	28 (49.12)	30 (52.63)	χ² = 0.14	0.710
	Unemployed	29 (50.88)	27 (47.37)		

Clinically significant depressive symptoms (PHQ-9 ≥10) were significantly more frequent among participants with T2DM compared with non-diabetic controls (Table 2). In the T2DM group, 35 participants (61.40%) screened positive for clinically significant depressive symptoms, whereas 19 participants (33.33%) in the control group screened positive. Conversely, 22 participants (38.60%) with T2DM and 38 participants (66.67%) from the control group did not screen positive for clinically significant depressive symptoms. The difference in the proportion of participants screening positive for clinically significant depressive symptoms between the two groups was statistically significant (χ² = 8.99, p = 0.003).

**Table 2:** Comparison of Clinically Significant Depressive Symptoms Between Adults With and Without T2DM (N = 114) Clinically significant depressive symptoms were defined as PHQ-9 score ≥10. OR represents the odds of screening positive among T2DM participants compared with controls. T2DM: Type 2 diabetes mellitus

Depression status (PHQ-9 ≥10)	T2DM (n=57) n (%)	Controls (n=57) n (%)	Effect estimate	Relative risk	Risk difference	χ²	p-value
Screened positive for clinically significant depressive symptoms	35 (61.40)	19 (33.33)	OR = 3.18 (95% CI: 1.48–6.85)	1.84	28.1%	8.99	0.003
Screened negative for clinically significant depressive symptoms	22 (38.60)	38 (66.67)					

Among participants with clinically significant depression (T2DM: n = 35; controls: n = 19), depression was more frequent in male participants (T2DM: 19, 54.29%; controls: 9, 47.37%) than female participants (T2DM: 16, 45.71%; controls: 10, 52.63%), with a significant association (p = 0.624), shown in Table 3. Across age groups, the highest proportion of depression was observed in the 46-50 years’ category in both T2DM (10, 28.57%) and controls (4, 21.05%), showing a significant trend (p = 0.790). Regarding severity, moderate depression was most frequent in both groups (T2DM: 20, 57.14%; controls: 16, 84.21%), followed by moderately severe and severe categories.

**Table 3:** Distribution of Demographic Characteristics and Depression Severity Among Participants Screening Positive for Clinically Significant Depressive Symptoms Percentages in the first three sections were calculated among participants screening positive for clinically significant depressive symptoms (T2DM: n=35; controls: n=19). Duration analysis was performed among diabetic participants only (n=35), according to depressive symptom status. Comparisons were performed using the chi-square test.

Variable	Category	T2DM (n = 35) n (%)	Controls (n = 19) n (%)	χ²	p-value
Gender	Male	19 (54.29)	9 (47.37)	0.24	0.624
	Female	16 (45.71)	10 (52.63)		
Age group (years)	30–35	6 (17.14)	4 (21.05)	1.05	0.790
	36–40	8 (22.86)	5 (26.32)		
	41–45	11 (31.43)	6 (31.58)		
	46–50	10 (28.57)	4 (21.05)		
Depression severity (PHQ-9)	Moderate (10–14)	20 (57.14)	16 (84.21)	4.34	0.114
	Moderately severe (15–19)	10 (28.57)	2 (10.53)		
	Severe (20–27)	5 (14.29)	1 (5.26)		
Duration of T2DM (n=57)	<1 year	9 (25.71)	—	7.23	0.027
	1–5 years	11 (31.43)	—		
	>5 years	15 (42.86)	—		

Multivariable binary logistic regression analysis demonstrated that T2DM was independently associated with clinically significant depressive symptoms after adjusting for potential confounding variables (Table 4). Participants with T2DM had 3.15-fold higher odds of screening positive for clinically significant depressive symptoms compared with non-diabetic controls (aOR = 3.15, 95% CI: 1.36-7.28, p = 0.009). Higher BMI was also independently associated with increased odds of depressive symptoms (aOR = 1.09 per kg/m² increase, 95% CI: 1.01-1.18, p = 0.039). Among patients with T2DM, a diabetes duration of more than 5 years was significantly associated with depressive symptoms compared with a duration of less than one year (aOR = 4.52, 95% CI: 1.09-18.71, p = 0.044). In contrast, age, sex, residence, education level, socioeconomic status, employment status, and diabetes duration of 1-5 years were not independently associated with clinically significant depressive symptoms after adjustment (p > 0.05 for all). These findings indicate that T2DM, increased BMI, and longer diabetes duration are independent predictors of clinically significant depressive symptoms in the study population.

**Table 4:** Multivariable Logistic Regression Analysis of Factors Associated With Clinically Significant Depressive Symptoms (PHQ-9 ≥10) (N =114) Dependent variable: clinically significant depressive symptoms (PHQ-9 ≥10; yes/no). Variables entered into the model included diabetes status, age, sex, BMI, residence, education level, socioeconomic status, employment status, and duration of T2DM. aOR: adjusted odds ratio; CI: confidence interval; Wald χ²: Wald chi-square statistic. Reference categories are indicated for categorical variables.

Variable	Category	Adjusted OR (aOR)	95% CI	Wald χ²	p-value
Diabetes status	Control (Reference)	1.00	Reference	—	—
	T2DM	3.15	1.36–7.28	6.89	0.009
Age (years)	Continuous	1.04	0.99–1.10	1.71	0.191
Gender	Male (Reference)	1.00	Reference	—	—
	Female	1.18	0.51–2.72	0.16	0.689
BMI (kg/m²)	Continuous	1.09	1.01–1.18	4.24	0.039
Residence	Urban (Reference)	1.00	Reference	—	—
	Rural	1.12	0.49–2.58	0.08	0.774
Education	Primary (Reference)	1.00	Reference	—	—
	Secondary	0.86	0.31–2.41	0.09	0.765
	Higher secondary	0.74	0.26–2.15	0.29	0.591
Socioeconomic status	Low (Reference)	1.00	Reference	—	—
	Middle	0.82	0.34–1.99	0.20	0.655
	High	0.63	0.21–1.89	0.63	0.427
Employment status	Employed (Reference)	1.00	Reference	—	—
	Unemployed	1.37	0.61–3.09	0.57	0.450
Duration of T2DM	<1 year (Reference)	1.00	Reference	—	—
	1–5 years	1.81	0.48–6.81	0.78	0.377
	>5 years	4.52	1.09–18.71	4.05	0.044

## Discussion

The present study found that patients with T2DM were significantly more likely to be depressed than patients who were not diabetic. The diabetic patients had 61.40% (n = 35) who were depressed, whereas the non-diabetic patients had 33.33% (n = 19), which was statistically significantly associated (χ² = 8.99, p = 0.003). The results of the present study are similar to the previous studies conducted in Pakistan and elsewhere, which reported the prevalence of depression among diabetic patients to be between 38% and 60%. In the Pakistani population, for example, Arshad et al. (2016) reported a depression prevalence of 38.3% in T2DM patients, and Sharif et al. (2023) found a prevalence rate of over 50% in multicenter cohorts [15,16]. This may be because of lifestyle constraints and poor mental health screening in outpatient diabetic management that have led to a higher psychological burden in our study.

The current study showed that diabetic patients had a significantly higher BMI (27.84 ± 4.12 kg/m²), which may partly explain the greater burden of depression reported in previous studies. This is in line with what has been reported in the international literature, as obesity and insulin resistance are closely related to depressive symptomatology both through inflammatory and neuroendocrine pathways. It has been shown that an increased BMI in diabetic patients is associated with poorer psychological functioning and lower quality of life, thus confirming the metabolic-psychological interaction in T2DM patients [17].

Stratified analysis in our study showed no statistically significant difference in the prevalence of clinically significant depressive symptoms between male and female participants within the T2DM group. Although previous studies have often reported a higher burden of depression among women with diabetes, our findings did not demonstrate a significant gender-based difference. Zahid et al. [18] reported a greater prevalence of depression among female patients with diabetes than male patients in a rural Pakistani population, while Perveen et al. [19] also evaluated gender distribution among adults with newly diagnosed T2DM but did not identify sex as an independent predictor of depression after adjustment for potential confounders. The discrepancy between our findings and previous reports may be attributable to differences in study populations, sample size, diabetes duration, assessment methods, and sociodemographic characteristics. Overall, our results suggest that both male and female patients with T2DM are vulnerable to depressive symptoms, highlighting the importance of routine depression screening regardless of sex.

The age-stratified analysis suggested a higher proportion of participants screening positive for clinically significant depressive symptoms in the 46-50-year age group compared with younger age categories. However, given the relatively small sample size within individual age strata, these subgroup findings should be interpreted with caution. The overall trend is consistent with previous studies reporting that middle-aged adults with T2DM are more likely to experience depressive symptoms because of the cumulative burden of living with diabetes, increasing risk of chronic complications, and longer disease duration. A study conducted in Karachi similarly reported a positive association between increasing age and the severity of depressive symptoms among individuals with diabetes, reflecting the progressive psychological burden associated with advancing age and prolonged illness [19]. Nevertheless, larger adequately powered studies are required to confirm these age-related differences.

In addition, our study found no statistically significant difference in the distribution of depression severity (moderate, moderately severe, and severe) between participants with T2DM and non-diabetic controls (p = 0.114). Moderate depression was the most common category in both groups, accounting for 57.14% of diabetic participants and 84.21% of controls with clinically significant depressive symptoms. A similar predominance of moderate depressive symptoms among individuals with T2DM has been reported in Pakistani studies using the PHQ-9, including the recent study by Fatima et al. [20]. Earlier studies from Pakistan have likewise demonstrated a high burden of depression among people with diabetes, although differences in study populations, diagnostic thresholds, and assessment tools have resulted in variable estimates of severity and prevalence [18,19]. These findings suggest that while T2DM is associated with a higher likelihood of clinically significant depressive symptoms, the pattern of depression severity among affected individuals may be broadly comparable between diabetic and non-diabetic populations.

Lastly, the duration of diabetes was significantly associated with depressive symptoms among participants with T2DM. The highest proportion of clinically significant depressive symptoms was observed in patients with a diabetes duration of >5 years (42.86%), followed by those with 1-5 years (31.43%) and <1 year (25.71%) (p = 0.027). Furthermore, multivariable logistic regression demonstrated that a diabetes duration of more than five years independently increased the odds of clinically significant depressive symptoms (aOR = 4.52, 95% CI: 1.09-18.71, p = 0.044). These findings are consistent with Kim et al., who reported that longer diabetes duration was independently associated with greater depressive symptom scores, particularly among elderly men with T2DM [21]. The observed relationship may reflect the cumulative psychological burden of long-term disease management, progressive metabolic dysregulation, treatment fatigue, and the increased likelihood of developing diabetes-related complications over time.

Study strength and limitations

The present study has several strengths. It employed a well-defined comparative cross-sectional design with equal numbers of participants in the T2DM and control groups, which minimized selection imbalance and enhanced the internal validity of the comparisons. Depression was assessed using the validated Patient Health Questionnaire-9 (PHQ-9), ensuring standardized and consistent measurement across both groups. Furthermore, the collection of detailed demographic and clinical information, including age, sex, BMI, socioeconomic status, and duration of diabetes, enabled stratified analyses that provided additional insights into factors associated with depression among adults with T2DM.

Several limitations should be acknowledged. First, participants were recruited using non-probability consecutive sampling from the outpatient department of a single tertiary care hospital, which may have introduced selection bias and limited the external validity of the findings. Therefore, the results should be interpreted as representative of a hospital-based outpatient population rather than the general Pakistani adult population or community prevalence of depression. Second, the cross-sectional design precludes establishing a temporal or causal relationship between T2DM and depression. Third, although the PHQ-9 is a validated screening instrument, it does not replace a comprehensive clinical psychiatric evaluation, and some degree of misclassification may have occurred. Finally, potentially important confounding factors, such as physical activity, dietary habits, social support, stressful life events, and medication adherence, were not comprehensively assessed or adjusted for and may have influenced the observed associations. Future multicenter, community-based longitudinal studies employing probability sampling and diagnostic psychiatric assessments are recommended to improve the generalizability and strengthen causal inference.

## Conclusions

The study shows that psychological morbidity is significantly associated with metabolic disease as depression is more common in people with T2DM than in non-diabetics. The distribution of the severity of depression is not significantly different across groups, but the overall burden is greater in the diabetic population, underscoring the need for ongoing mental health screening of diabetic patients. Additionally, depression shows an association with a longer duration of diabetes. Longitudinal cohort studies need to be conducted to improve the temporal and causal relationship between T2DM and depression. To enhance generalizability, larger multicenter studies with probability sampling are recommended. The use of structured psychiatric diagnostic interviews in addition to screening instruments would improve diagnostic accuracy, and the incorporation of psychosocial and lifestyle factors could help to identify modifiable risk factors and target mental health interventions in diabetic populations.

## Appendices

**Table 5:** Structured Data Collection Questionnaire Used in the Study T2DM: Type 2 diabetes mellitus; PHQ-9: Patient Health Questionnaire-9

Section	Variable	Response Options/Measurement
A. Participant Identification	Participant ID	
	Date of Interview	
	Study Group	□ Type 2 Diabetes Mellitus (T2DM) □ Non-diabetic Control
B. Informed Consent	Written informed consent obtained	□ Yes □ No (If No, exclude)
C. Demographic Characteristics	Age (years)	
	Age Category	□ 30–35 □ 36–40 □ 41–45 □ 46–50
	Gender	□ Male □ Female
	Place of Residence	□ Urban □ Rural
	Educational Level	□ Primary □ Secondary □ Higher Secondary
	Socioeconomic Status	□ Low □ Middle □ High
	Employment Status	□ Employed □ Unemployed
D. Anthropometric Measurements	Height (cm)	
	Weight (kg)	
	Body Mass Index (kg/m²)	
E. Diabetes Assessment	Previous physician diagnosis of Type 2 Diabetes Mellitus	□ Yes □ No
	Diagnosis confirmed through medical records	□ Yes □ No
	Current glucose-lowering medication	□ Yes □ No
	Duration of T2DM	□ <1 year □ 1–5 years □ >5 years
F. Eligibility Screening (Exclusion Criteria)	Previously diagnosed psychiatric disorder	□ Yes □ No
	Currently receiving psychiatric treatment	□ Yes □ No
	Hypertension	□ Yes □ No
	Chronic Kidney Disease	□ Yes □ No
	Liver Cirrhosis	□ Yes □ No
	Stroke	□ Yes □ No
	Other endocrine disorders (except T2DM)	□ Yes □ No
	Substance abuse	□ Yes □ No
	Alcohol abuse	□ Yes □ No
	Eligible for inclusion	□ Yes □ No
G. Depression Assessment (PHQ-9)	PHQ-9 administered	□ Yes □ No
	PHQ-9 Total Score	______ (0–27)
	Depression Category	□ None (0–4) □ Mild (5–9) □ Moderate (10–14) □ Moderately Severe (15–19) □ Severe (20–27)
	Clinically significant depressive symptoms (PHQ-9 ≥10)	□ Yes □ No
H. Data Used for Statistical Analysis	Depression status	□ Positive (PHQ-9 ≥10) □ Negative (PHQ-9 <10)
	Depression severity (if PHQ-9 ≥10)	□ Moderate □ Moderately Severe □ Severe
	Group allocation	□ T2DM □ Control
	BMI entered for multivariable analysis	______ kg/m²
	Age entered for multivariable analysis	______ years
	Gender entered for multivariable analysis	□ Male □ Female
	Residence entered for multivariable analysis	□ Urban □ Rural
	Education entered for multivariable analysis	□ Primary □ Secondary □ Higher Secondary
	Socioeconomic status entered for multivariable analysis	□ Low □ Middle □ High
	Employment status entered for multivariable analysis	□ Employed □ Unemployed
	Duration of T2DM entered for multivariable analysis (T2DM only)	□ <1 year □ 1–5 years □ >5 years
